# Prevalence of mixed neuropathologies in age‐related neurodegenerative diseases: A community‐based autopsy study in China

**DOI:** 10.1002/alz.14369

**Published:** 2024-11-25

**Authors:** Xiaoxi Wang, Keqing Zhu, Wei Wu, Dan Zhou, Hui Lu, Juan Du, Li Cai, Xiaoxin Yan, Wensheng Li, Xiaojing Qian, Xue Wang, Chao Ma, Yuting Hu, Chen Tian, Bing Sun, Zheng Fang, Juanli Wu, Peiran Jiang, Jianxin Liu, Cuiyun Liu, Jiayao Fan, Huixian Cui, Yi Shen, Shumin Duan, Aimin Bao, Ying Yang, Wenying Qiu, Jing Zhang

**Affiliations:** ^1^ Department of Pathology The First Affiliated Hospital Zhejiang University School of Medicine Hangzhou China; ^2^ National Health and Disease Human Brain Tissue Resource Center School of Medicine Zhejiang University Hangzhou China; ^3^ Department of Neurobiology and Department of Neurology of the Second Affiliated Hospital Zhejiang University School of Medicine Hangzhou China; ^4^ Institute of Basic Medical Sciences Neuroscience Center National Human Brain Bank for Development and Function Chinese Academy of Medical Sciences Beijing China; ^5^ Department of Human Anatomy Histology and Embryology School of Basic Medicine Peking Union Medical College Beijing China; ^6^ School of Public Health and the Second Affiliated Hospital Zhejiang University School of Medicine Hangzhou China; ^7^ Fudan branch of National Health and Disease Human Brain Tissue Resource Center Department of Human Anatomy and Histoembryology School of Basic Medical Sciences Fudan University Shanghai China; ^8^ Department of Human Anatomy Human Brain Bank Hebei Medical University Shijiazhuang China; ^9^ Department of Pathology School of Basic Medicine Anhui Medical University Hefei China; ^10^ Department of Anatomy & Neurobiology Central South University Xiangya School of Medicine Changsha China; ^11^ Center for Basic and Translational Research The 2nd Affiliated Hospital Zhejiang University School of Medicine Hangzhou China; ^12^ Department of Mental Health The First Affiliated Hospital Zhejiang University School of Medicine Hangzhou China; ^13^ Department of Neurobiology and Department of General Intensive Care Unit of the Second Affiliated Hospital Zhejiang University School of Medicine Hangzhou China; ^14^ NHC and CAMS Key Laboratory of Medical Neurobiology MOE Frontier Science Center for Brain Research and Brain‐Machine Integration Zhejiang University School of Brain Science and Brain Medicine Hangzhou China; ^15^ Research Units for Emotion and Emotion Disorders Chinese Academy of Medical Sciences Hangzhou China; ^16^ Department of Psychiatry Sir Run Run Shaw Hospital Zhejiang University School of Medicine Hangzhou China

**Keywords:** aging‐related tau astrogliopathy, Alzheimer's disease neuropathological change, cerebrovascular disease, Lewy body disease, limbic‐predominant age‐related TDP‐43 encephalopathy, mixed neuropathology, primary age‐related tauopathy

## Abstract

**INTRODUCTION:**

Despite extensive studies on mixed neuropathologies, data from China are limited. This study aims to fill this gap by analyzing brain samples from Chinese brain banks.

**METHODS:**

A total of 1142 brains from six Chinese brain banks were examined using standardized methods. Independent pathologists conducted evaluations with stringent quality control. Prevalence and correlations of neurological disorders were analyzed.

**RESULTS:**

Significant proportions of brains displayed primary age‐related tauopathy (PART, 35%), limbic‐predominant age‐related TDP‐43 encephalopathy (LATE, 46%), and aging‐related tau astrogliopathy (ARTAG, 12%). Alzheimer's disease neuropathological change (ADNC, 48%), Lewy body disease (LBD, 13%), and cerebrovascular disease (CVD, 63%) were also prevalent, often co‐occurring with regional variations. CVD emerged as the potential most early contributor to neuropathological changes.

**DISCUSSION:**

This analysis highlights the prevalence of PART, LATE, ARTAG, ADNC, LBD, and CVD, with regional differences. The findings suggest CVD may be the earliest contributing factor, potentially preceding other neuropathologies.

**Highlights**

- The prevalence of primary age‐related tauopathy (PART), limbic‐predominant age‐related TDP‐43 encephalopathy (LATE), aging‐related tau astrogliopathy (ARTAG), Alzheimer's disease neuropathologic change, Lewy body disease, and cerebrovascular disease (CVD) in China, increasing with age, is comparable to other countries.
- Significant regional differences in the prevalences of diseases are noted.
- CVD develops prior to any other disorders, including PART, LATE, and ARTAG.

## 1 BACKGROUND

Older adults, whether symptomatic or asymptomatic, frequently exhibit a mix of neurodegenerative pathologies in their brains, known as mixed pathologies.^1^, ^2^ The most common types include Alzheimer's disease (AD), characterized by amyloid beta (Aβ) plaques and neurofibrillary tangles (NFTs) with hyperphosphorylated tau; Lewy body disease (LBD), marked by α‐synuclein–containing Lewy bodies; and various cerebrovascular diseases (CVD).^1^

More recent studies on aging frequently report vascular injuries, as well as intricate combinations of age‐related and incidental pathologies associated with the accumulation of Aβ, tau, α‐synuclein, and TDP‐43 proteins.^1^ Primary age‐related tauopathy (PART) is a tauopathy characterized by NFTs but without significant Aβ plaques, typically observed in elderly individuals.^3^ Limbic‐predominant age‐related TDP‐43 encephalopathy (LATE) is characterized by TDP‐43 proteinopathy, largely affecting the limbic system, particularly the hippocampus and amygdala.^4^ A review indicates that aging‐related tau astrogliopathy (ARTAG) is an age‐associated tau pathology that primarily affects astrocytes.^5^

A significant observation regarding mixed pathology and age‐related brain pathologies is the variation in disease prevalence and geographic distribution. In developed countries, the prevalence of co‐occurring diseases varies notably across different European regions. One study reported that the prevalence of pathologies was up to 68% for AD neuropathological change (ADNC), 38% for ARTAG, 75% for TDP‐43 pathology, 39% for LBD pathology, and up to 70% for vascular pathology.^6^ Another study found a similar prevalence for ADNC (68.7%), but a lower prevalence for LBD pathology (24.9%) and vascular lesions (48.9%), and a much lower prevalence for TDP‐43 proteinopathy (13.3%).^7^ A more recent study in Northern Europe found that the prevalence rates were 35% for LATE, 39% for ADNC, and 59% for PART.^8^ Importantly, data on mixed pathology or age‐related disorders in populations other than those of European ancestry, such as African Americans, Hispanics, and Asians, are notably lacking.^6^

In Asia, data on mixed pathology are rather limited. A Japanese study of forensic autopsy cases found that among patients with argyrophilic grain disease (AGD), 23.4% had dementia, and 15.2% had a history of psychiatric hospital visits.^9^ A study from a Chinese human brain bank showed that 12% (21 out of 180) *post mortem* brains were neuropathologically diagnosed as LBD, without reporting any mixed pathology results.^10^

To address the dearth of comprehensive brain pathology studies in China and Asia, this study conducts a large‐scale, autopsy‐based analysis across multiple Chinese brain banks. By applying standardized sampling and staining protocols to a substantial dataset from six geographically diverse regions, we investigate the prevalence of age‐related brain pathologies, major neurodegenerative diseases, and their interconnections.

## 2 METHODS

### 2.1 Research samples

The study was conducted following the Declaration of Helsinki^11^ and approved by the institutional review board of Zhejiang University School of Medicine, Hangzhou, Zhejiang, China (2020‐005). Participants gave written informed consent. Over a 12‐year span from 2012 to early 2024, a comprehensive neuropathological assessment was conducted on 1142 cases across the China brain banks, located in northern (Peking Union Medical College and Hebei Medical University) and southern (Zhejiang University, Central South University, Fudan University, and Anhui Medical University) regions. Each case underwent a thorough clinical autopsy, with comprehensive medical records available for subsequent analysis.

### 2.2 Immunohistochemistry

Immunohistochemistry (IHC) analysis was conducted on formalin‐fixed brain tissues from all autopsy cases. Following the established protocol,^12^ donated brains were immersed in 10% buffered formalin in a 0.1 mol/L phosphate‐buffered saline solution (pH 7.4). Small blocks of brain tissue were then excised from targeted regions, fixed in formalin for 2 days, dehydrated, and embedded in paraffin.

Using a microtome, the paraffin‐embedded brain tissue was sliced into 6‐µm thick sections. To minimize endogenous peroxidase activity, the sections were treated with 3% H_2_O_2_ for 15 minutes. Antigen retrieval was performed via microwave irradiation in citrate buffer (pH 6.0) for 15 minutes. Before incubation with the primary antibodies, the sections were blocked with 10% normal goat serum to prevent non‐specific binding.

The primary antibodies used in this study included anti‐tau AT8 (hyperphosphorylated tau, mouse monoclonal, 1:200, Thermo Fisher Scientific), anti‐Aβ (mouse monoclonal, 1:200, Sigma‐Aldrich), anti‐pTDP‐43 (rabbit polyclonal, 1:600, Sigma‐Aldrich), and anti‐α‐synuclein (mouse monoclonal, clone KM51, 1:50, Leica). These antibodies were incubated with the sections overnight at 4°C. After thorough washing, the sections were exposed to the appropriate secondary antibody and the avidin‐biotinylated horseradish peroxidase (HRP) complex (ABC) system. The secondary antibody systems used were sourced from the same company (PV‐6001, PV‐6002, ZSGB‐BIO, China) across all participating centers, and the staining was performed manually.

To visualize the immunoreactivity, HRP activity was detected using a solution containing 0.4 mg/mL 3,3′‐diaminobenzidine (DAB) and 0.0006% hydrogen peroxide in Tris‐buffered saline (TBS). For negative control sections, only the secondary antibody and the avidin‐biotinylated HRP complex were used. After alignment on gelatin‐coated slides and drying, the sections underwent dehydration and clearing procedures. Finally, the sections were mounted with a suitable medium for further analysis.

**RESEARCH IN CONTEXT**

1. **Systematic review**: The authors reviewed the literature using traditional sources (e.g., PubMed). While there are data on comorbid pathology or age‐related disorders in populations of European ancestry, information on comorbidities in other populations such as African Americans, Hispanics, and Asians, is notably lacking. These relevant references are appropriately cited.
2. **Interpretation**: This study leverages a comprehensive autopsy‐based analysis of brain banks across China, using standardized sampling and staining protocols. The study identified primary age‐related tauopathy, limbic‐predominant age‐related TDP‐43 encephalopathy, aging‐related tau astrogliopathy, Alzheimer's disease neuropathologic change, Lewy body disease, and cerebrovascular disease (CVD) as commonly diagnosed neuropathological conditions in the study population of China, with varying rates of co‐occurrence and distinct regional distribution. An association study indicates that CVD likely represents the earliest alterations in all diseases investigated.
3. **Future directions**: This largest community‐based autopsy study in China and Asia highlights the substantial prevalence and co‐occurrence of diverse neuropathologies, addressing a significant gap in current research. However, our study has several limitations. Future research should address these limitations by including a more ethnically diverse sample, conducting thorough analyses of clinical cognitive correlations, and considering the impact of apolipoprotein E ε4 gene status.

### 2.3 Neuropathological assessment

During the autopsy procedure, the brains underwent a series of rigorous evaluations. Initially, the brains were weighed and immersed in 10% buffered formalin for at least 2 weeks. After this, the brains were sliced into 0.5 cm–thick coronal sections for further examination. Macroscopic lesions and vascular abnormalities were carefully assessed, and standardized brain samples were collected from 22 distinct regions, as outlined in Table S1 in supporting information. Subsequently, additional IHC stains were applied to the brain samples, with the specific stains summarized in Table S1.

Notably, our study used uniformly defined assessment criteria across various Chinese brain banks to ensure consistency and reliability. The IHC results were evaluated in accordance with the recommended staging systems for hyperphosphorylated‐tau Braak stage, Aβ Thal phase, αS BrainNet Europe (BNE) stage,^13^ and TDP‐43 regions.^4^ The presence and severity of ADNC were evaluated using the “ABC” scoring system, which classifies changes as “low” (L), “intermediate” (I), or “high” (H) ADNC.^5^, ^7^ The PART subjects were identified based on stringent criteria, including an NFT Braak stage of ≤ IV and Thal Aβ Phase 0 to 1.^3^ The spectrum of vascular pathologies evaluated ranged from large macroscopic and smaller microscopic infarcts, lacunar infarcts, hemorrhages, atherosclerosis, arteriosclerosis, to cerebral amyloid angiopathy (CAA).^14^, ^15^ A detailed description of the CVD assessment is provided in Table S2 in supporting information. LBD, including Parkinson's disease (PD) and incidental PD, was diagnosed based on the positive detection of α‐synuclein by pathology, even in cases without clinical manifestations of PD.^16^, ^17^, ^18^, ^19^ ARTAG was diagnosed in accordance with the harmonized evaluation strategy.^5^

To validate the diagnosis, we collaborated with the Netherlands Brain Bank and conducted a staining comparison to our own brain banks. The analysis revealed that all results were in alignment with the diagnoses established by the Netherlands Brain Bank, confirming the accuracy of our assessment. To further standardize the procedures, we shared a subset of cases in a blinded manner to assess inter‐rater variability in the evaluation of ADNC levels. For questionable cases, we used multi‐headed microscopes to enhance accuracy. Representative stained images of tau AT8, Aβ, pTDP‐43, and α‐synuclein, as well as CAA, ARTAG, infarct, and hemorrhage, are provided in Figure S1 in supporting information.

### 2.4 Statistical analysis

The prevalence and mixed pathology of neuropathological brain disorders were analyzed. National consolidated metrics were estimated by pooling all samples across the brain banks. Additionally, the regional prevalence and mixed pathology in the northern and southern brain banks were evaluated. The correlation between each pair of neuropathological conditions was estimated using the Phi coefficient (*φ*), which measures the mean square contingency coefficient. The statistical significance of the correlation between each neuropathological brain disorder was assessed by the chi‐square test, followed by regression analysis to adjust for age, sex, and Braak NFT stage. All statistical analyses were performed using R version 4.2.2.

## 3 RESULTS

### 3.1 The general demographic characteristics of donated brains

**FIGURE 1:**
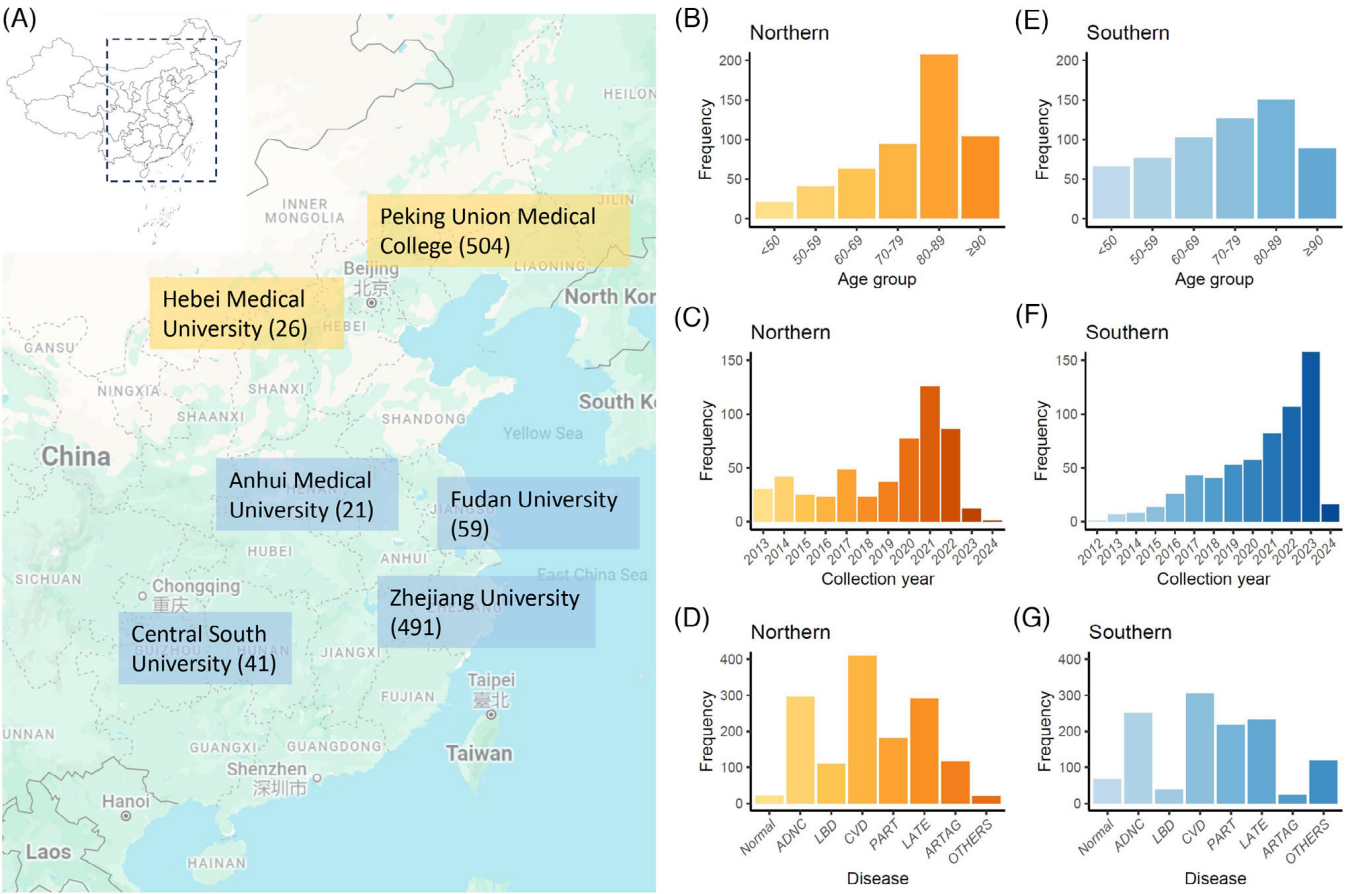
The general information of the 1142 donated brains from six national brain banks. A, Based on the geographical location, the brain banks were categorized into northern brain banks, which include Peking Union Medical College and Hebei Medical University, and southern brain banks, which include Zhejiang University Brain Bank, Central South University Brain Bank, Fudan University Brain Bank, and Anhui Medical University Brain Bank. The northern and southern banks are colored yellow and blue, respectively. B, The age distributions of the donated brains in northern brain banks. C, The distributions of collection years in northern brain banks. D, The number of samples with neuropathological disorder diagnoses in northern brain banks. E, The age distributions of the donated brains in southern brain banks. F, The distributions of collection years in southern brain banks. G, The number of samples with neuropathological disorder diagnoses in southern brain banks.

This study included 1142 donated brains from six national brain banks (Figure 1A), consisting of 719 males (63%) and 423 females (37%; Table 1). The age of the samples ranged from 18 to 106 years, with a median age of 79 years, and interquartile ranges of 65 (P25) to 87 (P75) years. Specifically, 87 brains were from individuals younger than 50 years, with 57 males (66%) and 30 females (34%). Additionally, 25 donors were aged 18 to 30 years, with 5 donors from the northern brain Banks and 20 from the southern brain banks (Table S3 in supporting information). In the age group of 50 to 59 years, there were 118 brains (68 males [58%] and 50 females [42%])); for the age group of 60 to 69 years, there were 166 brains (115 males [69%] and 51 females [31%]); 221 brains were aged 70 to 79 years (157 males [71%] and 64 females [29%]); 357 brains were aged 80 to 89 years (205 males [57%] and 152 females [43%]); and 193 brains were 90 years or older (117 males [61%] and 76 females [39%]).

**TABLE 1:** Demographics of all brain tissue donors.

Age groups (years)	*N*	Sex		Normal	ADNC	LBD	CVD	PART	LATE	ATARG	Others
		M	F								
< 50	87	57 (66)	30 (34)	42 (48)	2 (2)	1 (1)	21 (24)	6 (7)	2 (2)	2 (2)	14 (16)
50–59	118	68 (58)	50 (42)	26 (22)	17 (14)	3 (3)	48 (41)	44 (37)	16 (14)	0 (0)	21 (18)
60–69	166	115 (69)	51 (31)	15 (9)	52 (31)	10 (6)	86 (52)	84 (51)	41 (25)	6 (4)	22 (13)
70–79	221	157 (71)	64 (29)	5 (2)	109 (49)	28 (13)	140 (63)	96 (43)	105 (48)	19 (9)	25 (11)
80–89	357	205 (57)	152 (43)	1 (0)	225 (63)	62 (17)	270 (76)	122 (34)	224 (63)	67 (19)	36 (10)
≥ 90	193	117 (61)	76 (39)	0 (0)	141 (73)	45 (23)	149 (77)	48 (25)	134 (69)	45 (23)	20 (10)
All subjects (%)	1142	719 (63)	423 (37)	89 (8)	546 (48)	149 (13)	714 (63)	400 (35)	522 (46)	139 (12)	138 (12)

Abbreviations: ADNC, Alzheimer's disease neuropathological change; ARTAG, aging‐related tau astrogliopathy; CVD, cerebrovascular disease; LATE, limbic‐predominant age‐related TDP‐43 encephalopathy; LBD, Lewy body disease; PART, primary age‐related tauopathy.

Based on the geographical location, the brain banks were categorized into northern brain banks (including Peking Union Medical College Brain Bank and Hebei Medical University Brain Bank) and southern brain banks (Zhejiang University Brain Bank, Central South University Brain Bank, Fudan University Brain Bank, and Anhui Medical University Brain Bank; Figure 1A). The northern brain banks had a concentration of samples primarily in the 80 to 89 age group, whereas the southern brain banks showed a relatively even distribution of samples across different age groups (Figure 1B,E, Tables S4 and S5 in supporting information). Since 2012, the northern brain banks have maintained a relatively consistent collection rate initially but experienced a significant increase in collections from 2020 to 2022. Meanwhile, the southern brain banks have seen a steady annual increase in the number of samples collected (Figure 1C**–**G). As expected, the proportion of normal brain tissue gradually decreased with advancing age (Figure S2 in supporting information, Table 1, and Tables S4 and S5).

### 3.2 The prevalence and mixed pathology of PART, LATE, and ARTAG

Among all the brains, 35% met the strict criteria for a conclusive diagnosis of PART (Table 1). This prevalence was consistent across both northern and southern regions (Tables S4 and S5). The prevalence of PART peaked in the 60 to 69 age group (Figure S2A), with similar patterns observed in both the northern and southern brain banks (Figure S2B,C). Among all the brains, 46% were diagnosed with LATE, with a prevalence of 55% in the northern brain banks and 38% in the southern brain banks. The prevalence of LATE increased with age, reaching as high as 69% among individuals aged ≥ 90 years. This trend was observed in both the northern and southern brain banks. As expected, a portion of LATE cases was associated with hippocampal sclerosis (HS; 30 out of 41 cases). Regarding ARTAG, 12% of the brains were diagnosed, with a prevalence of 22% in the northern brain banks and 4% in the southern brain banks. The prevalence of ARTAG also increased with age, reaching 23% among individuals aged ≥ 90 years (33% in the northern brain banks and 12% in the southern brain banks). When categorizing ARTAG into different subtypes for analysis, we found that most subtypes exhibited an age‐related increasing trend, with subpial and perivascular variants being the most prominent (Figure S3 in supporting information). The upward trends in gray matter and subependymal brain regions were more limited. These patterns were generally consistent across both the northern and southern brain banks, although the northern region showed a more noticeable increase due to higher prevalence.

Among the 1142 brains, 165 (14%) exhibited both PART and LATE, 43 (4%) exhibited both PART and ARTAG, and 97 (8%) exhibited both LATE and ARTAG (Table 2). There were 28 cases with concurrent PART, LATE, and ARTAG, comprising 18 males and 10 females. The median age of these 28 cases was 82.5 years, ranging from 65 to 98 years. To better illustrate the co‐occurrence of different pathologies, we created Venn diagrams of PART, LATE, and ARTAG across different age groups (Figure S4A–F in supporting information).

**TABLE 2:** Demographics of comorbidities in all brains.

	*N* (%)	Sex		ADNC	LBD	CVD	PART	LATE	ARTAG
		M	F						
ADNC	546	317 (58)	229 (42)	546 (100)	100 (18)	422 (77)	0 (0)	343 (63)	94 (17)
LBD	149	100 (67)	49 (33)	100 (67)	149 (100)	110 (74)	42 (28)	106 (71)	31 (21)
CVD	714	454 (64)	260 (36)	422 (59)	110 (15)	714 (100)	223 (31)	397 (56)	116 (16)
PART	400	278 (70)	122 (30)	0 (0)	42 (11)	223 (56)	400 (100)	165 (41)	43 (11)
LATE	522	325 (62)	197 (38)	343 (66)	106 (20)	397 (76)	165 (32)	522 (100)	97 (19)
ARTAG	139	86 (62)	53 (38)	94 (68)	31 (22)	116 (83)	43 (31)	97 (70)	139 (100)

Abbreviations: ADNC, Alzheimer's disease neuropathological change; ARTAG, aging‐related tau astrogliopathy; CVD, cerebrovascular disease; LATE, limbic‐predominant age‐related TDP‐43 encephalopathy; LBD, Lewy body disease; PART, primary age‐related tauopathy.

### 3.3 The prevalence and mixed pathology of CVD, ADNC, and LBD

**FIGURE 2:**
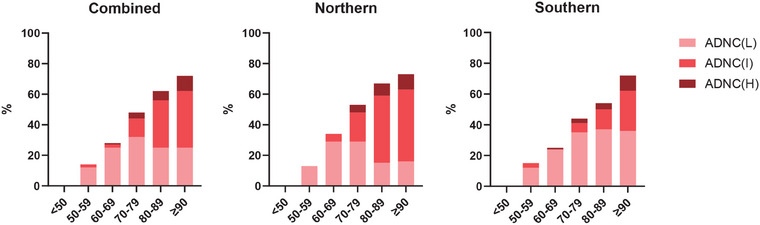
The prevalence and composition of different ADNC severity levels by age group. A, The prevalence and composition of different ADNC severity levels in all brains. B, The prevalence and composition of different ADNC severity levels in northern brain banks. C, The prevalence and composition of different ADNC severity levels in southern brain banks. ADNC was divided into three groups: low (L), intermediate (I), and high (H). The regional prevalence is detailed in Tables S4 and S5 in supporting information. ADNC, Alzheimer's disease neuropathological change.

CVD emerged as the most common pathological disorder, affecting 63% of the participants (77% in northern brain banks and 50% in southern brain banks). The prevalence of CVD increased with age, reaching 77% among individuals aged ≥ 90 years (82% in northern brain banks and 72% in southern brain banks). The prevalence of the CVD subcategories is detailed in Table 3, and their occurrence across different age groups is provided in Table S6 in supporting information. Demographic data for intracerebral hemorrhage and cerebral infarction are shown in Table S7 in supporting information. The data indicated that microhemorrhages predominated in cerebral hemorrhage, while microinfarcts were more common in cerebral infarction. The affected brain regions for both intracerebral hemorrhage and cerebral infarction were primarily concentrated in the brainstem and basal ganglia. Among the donors, ADNC was observed in 48% of the samples (56% in northern brain banks and 41% in southern brain banks, Table 1, Tables S4 and S5). As shown in Figure 2 and Figure S2, the prevalence of ADNC increased with age. In the group aged ≥ 90, 73% of the subjects were diagnosed with ADNC (72% in northern brain banks and 74% in southern brain banks). ADNC was further categorized into ADNC‐low, ADNC‐intermediate, and ADNC‐high subgroups. As shown in Figure 2, Table 4, Tables S8 and S9 in supporting information, the prevalence of ADNC‐I and ADNC‐H groups increased with age, while the prevalence of ADNC‐L peaked in the 70 to 79 age group. The prevalence of ADNC‐H group was 9% among individuals aged ≥ 90 years. LBD affected a smaller proportion of the subjects (13%), ranging from 6% in southern brain banks to 21% in northern brain banks (Table 1, Tables S4 and S5).

**TABLE 3:** Demographics of vascular disease.

	Total *N* (%)	Atherosclerosis	Arteriosclerosis	Intracerebral hemorrhage	Cerebral infarction	cerebral amyloid angiopathy (CAA)
All brain banks	714 (100)	406 (57)	291 (41)	114 (16)	244 (34)	220 (31)
Northern brain banks	409 (100)	328 (80)	140 (34)	58 (14)	159 (39)	141 (34)
Southern brain banks	305 (100)	78 (26)	151 (50)	56 (18)	85 (28)	79 (26)

**TABLE 4 alz14369-tbl-0004:** Demographics of ADNC subgroups in all brains.

Age groups (years)	*N*	ADNC	ADNC (L)	ADNC (I)	ADNC (H)
< 50	87	2 (2)	2 (2)	0 (0)	0 (0)
50–59	118	17 (14)	15 (13)	2 (2)	0 (0)
60–69	166	52 (31)	48 (29)	3 (2)	1 (1)
70–79	221	109 (49)	70 (32)	30 (14)	9 (4)
80–89	357	225 (63)	91 (25)	109 (31)	25 (7)
≥ 90	193	141 (73)	51 (26)	72 (37)	18 (9)
All subjects (%)	1142	546 (48)	277 (24)	216 (19)	53 (5)

Abbreviations: ADNC, Alzheimer's disease neuropathological change; H, high; I, intermediate; L, low.

**FIGURE 3 alz14369-fig-0003:**
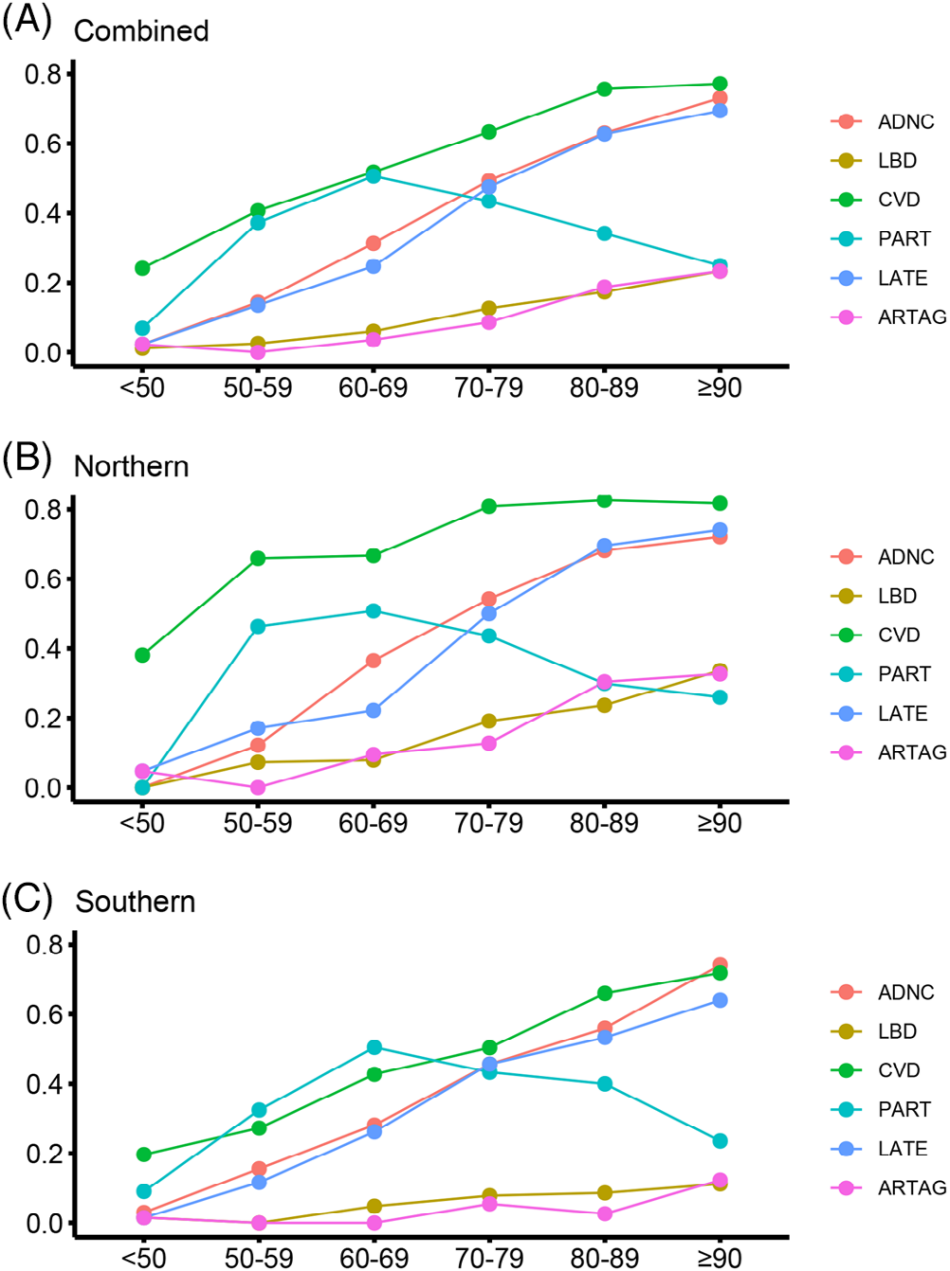
The prevalence of ADNC, LBD, CVD, PART, LATE, and ARTAG in each of the age groups. A, The prevalence of ADNC, LBD, CVD, PART, LATE, and ARTAG in all brains. B, The prevalence of ADNC, LBD, CVD, PART, LATE, and ARTAG in northern brain banks. C, The prevalence of ADNC, LBD, CVD, PART, LATE, and ARTAG in southern brain banks. ADNC, Alzheimer's disease neuropathological change; ARTAG, aging‐related tau astrogliopathy; CVD, cerebrovascular disease; LATE, limbic‐predominant age‐related TDP‐43 encephalopathy; LBD, Lewy body disease; PART, primary age‐related tauopathy.

When stratified by age, in each age subgroup, the prevalence of CVD was higher than that of ADNC and LBD (Figure 3, Table 1, and Tables S4 and S5). As early as age ≤ 50, the prevalence of CVD had reached 24%, while ADNC and LBD were almost non‐existent. The prevalence of ADNC increased year by year, nearly catching up with the prevalence of CVD in the ≥ 90 years age group.[Table alz14369-tbl-0004]

The coexistence of specific neuropathological conditions was also examined. Venn diagrams of ADNC, LBD, and CVD across different age groups are shown in Figure S4G–L. Among the 1142 brain samples, 422 cases (37%) exhibited both CVD and ADNC, 110 cases (10%) exhibited both CVD and LBD, and 100 cases (9%) exhibited both ADNC and LBD (Table 2). Additionally, 82 subjects simultaneously exhibited CVD, ADNC, and LBD, comprising 52 males and 30 females. The median age of these individuals was 87 years, with ages ranging from 60 to 102 years. The first quartile age was 82.8 years, and the third quartile age was 92.3 years. The occurrence of mixed pathologies became increasingly prevalent with advancing age.

### 3.4 Age‐related disorders and correlation with neuropathological conditions

The prevalence of mixed pathology using all brain samples[Fig alz14369-fig-0003] was statistically analyzed further. Venn diagrams of ADNC, LBD, CVD, PART, LATE, and ARTAG across different age groups are shown in Figure S4M–R. While PART and ADNC were diagnostically mutually exclusive, among the 400 PART cases, 223 (56%) exhibited concurrent CVD, 42 (11%) exhibited concurrent LBD, 165 (41%) exhibited concurrent LATE, and 43 (11%) exhibited concurrent ARTAG. In 522 LATE cases, 397 (76%) exhibited concurrent CVD, 343 (66%) exhibited concurrent ADNC, 106 (20%) exhibited concurrent LBD, and 97(19%) exhibited concurrent ARTAG. In 139 ARTAG cases, 116 (83%) exhibited concurrent CVD, 94 (68%) exhibited concurrent ADNC, 31 (22%) exhibited concurrent LBD, and 97 (70%) exhibited concurrent LATE (Table 2).

Analyzing from the perspective of three major neurological disorders, in 714 CVD cases, 223 (31%) exhibited concurrent PART, 397 (56%) exhibited concurrent LATE, and 116 (16%) exhibited concurrent ARTAG (Table 2). In 546 ADNC cases, 343 (63%) exhibited concurrent LATE, while 94 (17%) exhibited concurrent ARTAG. In 149 LBD cases, 42 (28%) exhibited concurrent PART, 106 (71%) exhibited concurrent LATE, and 31 (21%) exhibited concurrent ARTAG. Region‐specific results are detailed in Tables S10 and S11 in supporting information. Notably, in our study, several rare diseases were not included in the mixed pathology analysis: multiple system atrophy (MSA, *n* = 6), progressive supranuclear palsy (PSP, *n* = 7), frontotemporal dementia (FTD, *n* = 1), corticobasal degeneration (CBD, *n* = 1), and AGD (*n* = 3). Of note, the diagnosis of FTD referred to the clinical diagnosis rather than the neuropsychological diagnosis. Definitive chronic traumatic encephalopathy (CTE) was not identified in our collection.

**FIGURE 4:**
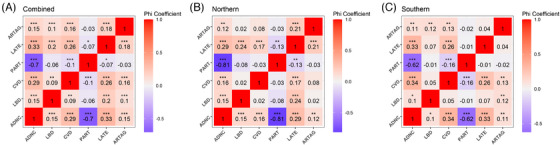
The association between age‐related pathological states (including PART, LATE, and ARTAG) and the diagnosis of ADNC, CVD, and LBD. A, The association between age‐related pathological states and the diagnosis of ADNC, CVD, and LBD in all brains. B, The association between age‐related pathological states and the diagnosis of ADNC, CVD, and LBD in northern brain banks. C, The association between age‐related pathological states and the diagnosis of ADNC, CVD, and LBD in southern brain banks. The correlation among neuropathological conditions was described by the φ coefficient. The significance of the correlation was estimated by the chi‐square test. ***, **, and * denote *p *< 0.001, *p *< 0.01, and *p *< 0.05, respectively. ADNC, Alzheimer's disease neuropathological change; ARTAG, aging‐related tau astrogliopathy; CVD, cerebrovascular disease; LATE, limbic‐predominant age‐related TDP‐43 encephalopathy; LBD, Lewy body disease; PART, primary age‐related tauopathy.

Next, the association between age‐related pathological states (PART, LATE, and ARTAG) and the diagnoses of CVD, ADNC, and LBD was investigated. We found that CVD started at a much earlier age than other conditions (Figure 3). The detection of CVD preceded ADNC and LBD. In the < 50 years age group, the detection rate of CVD exceeded 20%. In the 60 to 69 age group, half of the samples showed vascular lesions. As shown in Table S6, CVD lesions in younger donors were primarily characterized by cerebral hemorrhage, while the proportion of atherosclerosis and arteriolosclerosis increased with advancing age. Notably, in the northern region, the prevalence of CVD in elderly samples (< 70 years) was significantly higher than that in the southern region (Figure 3B,C). Meanwhile, the detection rate of ADNC in the northern region increased more rapidly with age, surpassing 30% in the 60 to 69 age group, compared to ≈ 20% in the southern region samples of the same age group. As shown in Figure 4A, CVD showed a stronger positive correlation with ADNC (*φ* = 0.29, *p* < 0.001) than with LBD (*φ* = 0.09, *p* < 0.01). After adjusting for age, sex, and Braak NFT stage (Figure S5 in supporting information), the association between CVD and ADNC remained significant (*p* < 0.001), whereas the association between CVD and LBD did not reach statistical significance (*p* > 0.05). We also estimated the associations between various CVD subtypes and neuropathological conditions, including ADNC, LBD, PART, LATE, and ARTAG. As shown in Figure S6 in supporting information, atherosclerosis was found to be associated with multiple pathological conditions, including ADNC, LBD, LATE, and ARTAG, with *φ* indices ranging from 0.15 to 0.26. Arteriosclerosis was found to be associated with ADNC, LATE, and ARTAG, with *φ* indices ranging from 0.12 to 0.19. CAA was associated with all pathological conditions, with *φ* indices ranging from –0.3 to 0.47. However, after adjusting for age, sex, and Braak NFT stage, the significant associations between atherosclerosis and ADNC/LBD, arteriosclerosis and ADNC/ARTAG, and CAA and LBD did not persist across all regions (Figure S7 in supporting information).

Correlation analysis also demonstrated that the detection of CVD preceded age‐related neuropathological conditions including PART, LATE, and ARTAG. Significant positive associations were observed between CVD and LATE (*φ* = 0.26, *p* < 0.001) and ARTAG (*φ* = 0.16, *p* < 0.001). Additionally, among the three age‐related conditions, significant associations were observed between ARTAG and LATE using the chi‐square test (*φ* = 0.18, *p *< 0.001, Figure 4). However, after adjusting for covariates, including age, sex, and Braak NFT stage, the significance was no longer present (*p* > 0.05, Figure S5). Notably, this pattern was consistent across all ARTAG subtypes (Figures S8 and S9 in supporting information). In addition, we estimated the associations between various ARTAG subtypes and neuropathological conditions, including ADNC, LBD, CVD, PART, and LATE. As shown in Figure S8, subpial and perivascular ARTAG were found to be associated with multiple pathological conditions, including ADNC, CVD, and LATE, with *φ* indices ranging from 0.08 to 0.15. However, after adjusting for age, sex, and Braak NFT stage, only the association between subpial ARTAG and CVD remained statistically significant (*p* < 0.01, Figure S9). Notably, white matter ARTAG was initially associated with ADNC (*φ* = 0.08, *p* < 0.01), but this association lost statistical significance after adjusting for covariates, including age, sex, and Braak NFT stage. Additionally, possibly due to the limited diagnosis of ARTAG in southern brain banks (Table S11), the association between ARTAG and LATE was only observed in northern brain banks (*φ* = 0.21, *p* < 0.001, Figure 4B,C). Significant associations were found between ARTAG and CVD pathologies using the chi‐square test (*p* < 0.001, Figure 4), and the significance persisted after adjusting for potential confounders (*p* < 0.05, Figure S5).

Finally, we analyzed age‐related conditions and two neurodegenerative diseases (Figure 4A). As expected, PART preceded ADNC before the age of 70. For samples aged ≥ 70, PART exhibited a negative correlation with ADNC (*φ* = –0.70, *p* < 0.001) due to their mutually exclusive nature of diagnoses. We observed a significant positive association between LATE and ADNC (*φ* = 0.33, *p* < 0.001) as well as LBD (*φ* = 0.20, *p* < 0.001). These associations remained statistically significant after covariate adjustment. ARTAG also showed significant positive associations with ADNC (*φ* = 0.15, *p* < 0.001) and LBD (*φ* = 0.10, *p* < 0.001), but these associations lost statistical significance after adjusting for age, sex, and Braak NFT stage, suggesting potential confounding factors for the association (Figure S5).

## 4 DISCUSSION

Our autopsy‐based neuropathological study, encompassing 1142 subjects (719 males, 423 females), is the largest of its kind in China and Asia. As expected, age‐related increases in PART, LATE, ARTAG, ADNC, LBD, and CVD were observed across most brain banks, with significant geographic variations between northern and southern regions. Notably, CVD pathology preceded all other conditions, including PART, LATE, and ARTAG, regardless of region.

PART typically occurs in the elderly without significant Aβ plaques. The prevalence of PART is 35% (34% in northern regions and 36% in southern regions), peaking in the 60 to 69 age group. The peak in the 60 to 69 age group is apparently related to the increase in the rate of Aβ pathologies in older age groups. The observed prevalence of PART, though it is similar between the northern and southern regions, is slightly higher than the previously reported prevalence. For instance, a study published in 2009 reported that NFT‐positive but neuritic plaque (NP)–negative cases comprise ≈ 5% of aged individuals across multiple datasets.^20^ In another study involving 233 individuals, the prevalence of non‐AD tauopathies (including novel forms) was 23.2%.^7^

LATE, another age‐related disorder, predominantly affects the elderly and primarily involves the limbic system, often associated with HS.^21^ In our investigation, 30 out of 41 LATE cases demonstrated HS. The average prevalence of LATE is 46%, peaking in individuals aged ≥ 90 years (69%), with a higher rate in the northern brain banks (55%) compared to southern brain banks (38%). Overall, the average prevalence is consistent with reports from other brain banks. Specifically, Nelson et al. reported that the prevalence of LATE increased with age, affecting ≈ 25% of individuals between 75 and 80 years old, and > 70% of individuals older than 100 years.^4^ Forrest et al. summarized that the prevalence of LATE varies widely, ranging from 13% to 75%.^6^

ARTAG, characterized by the deposition of tau protein in astrocytes, also primarily affects older adults.^22^ Forrest et al. showed the prevalence of ARTAG was 38%;^6^ Katsumata et al. indicated that 44.0% of individuals reported ARTAG.^22^ The prevalence of ARTAG in our cohort was 12%, with a higher prevalence in the northern regions (22%) compared to the southern regions (4%).

CVD refers to various conditions affecting the brain's vascular system, leading to interrupted or reduced blood supply to the brain, subsequently causing brain tissue damage and neurological dysfunction. In this study, the prevalence of CVD is 63% (77% in the northern region and 50% in the southern region), clearly showing a significant geographic difference. To this end, it is well documented that the prevalence of CVD in living patients is much higher in northern regions than in southern regions of China.^23^, ^24^, ^25^ However, the average prevalence of CVD at autopsy is comparable to other studies. For instance, according to a review by Forrest and Kovacs the prevalence of CVD ranges from 31% to 70% worldwide.^6^

ADNC refers to the various pathological features observed in the brains of patients with AD pathologies, that is, the simultaneous presence of NFTs and Aβ plaques. The prevalence of ADNC is 48% (56% in northern regions and 41% in southern regions). The prevalence of ADNC increases with age, reaching as high as 73% in individuals aged ≥ 90. The overall and age‐specific prevalence of ADNC is in line with the studies summarized recently by Forrest et al.^6^

LBD is a group of neurodegenerative diseases characterized by the presence of Lewy bodies in brain neurons or glia, which are primarily formed by the aggregation of α‐synuclein. The prevalence of LBD is 13% (21% in the northern and 6% in the southern regions), also in the range reported by others (6%–39%, depending on different countries).^6^

Previous investigations have indicated that mixed neuropathology was prevalent in the aging brain.^26^, ^27^ For example, a 2017 study by Josephs et al. reported that TDP‐43 proteinopathy was identified in ≈ 30% of cases with definite PART.^28^ Kovacs et al. showed that ARTAG was co‐present in 58.6% of PART cases and 62.6% of AD cases.^29^ Not surprisingly, our study found that, despite geographic differences, mixed neuropathology is common in Chinese populations: 165 out of 400 PART cases (41%) presented with LATE and 11% of PART cases, and 17% of ADNC cases were co‐present with ARTAG. Additionally, with increasing age, the prevalence of mixed pathologies and complexity escalated. This observation is consistent with the findings of a recently published study by Alafuzoff and Libard.^8^ However, Alafuzoff and Libard's study also analyzed cognitive impairments, a task not feasible in the current investigation due to the lack of detailed cognitive information for a significant portion of the donors. This issue must be addressed in future research. It should be noted also that the categories that differ significantly in the prevalences between the northern and southern brain banks include LATE, ARTAG, and CVD. While the underlying causes remain to be investigated, they may be related to genetic variation, dietary habits, and significant differences in weather conditions.

In addition to providing the basic prevalence of each major age‐related disorder, this relatively large‐scale brain bank offers an excellent opportunity to perform association studies. The association between PART and other diseases is tricky due to its non‐linear shape. Among other correlations, we observed that the detection of CVD was earlier than any other diseases, including PART, LATE, and ARTAG. Additionally, PART proceeded ADNC before the age of 70. Significant positive associations were observed between LATE and ARTAG, LATE and ADNC, as well as LATE and LBD. ARTAG also showed significant positive associations with ADNC and LBD. Further analysis revealed that several subtypes of ARTAG were associated with ADNC, CVD, and LATE. However, after adjusting for age, sex, and Braak NFT stage, most of these associations lost significance, partially consistent with recently reported findings.^22^

Clearly, a higher prevalence or association of disease A than or with B does not necessarily mean that disease A drives B. Yet, the fact that CVD was detected earlier than all other disorders might suggest that CVD is an important risk factor for diseases including PART, LATE, and ARTAG. Notably, CVD has been reported as a key risk factor for ADNC and LBD.^30^, ^31^, ^32^, ^33^, ^34^ To this end, the size and location of infarcts and hemorrhages are crucial for understanding the role of CVD in cognitive decline or movement disorders. Future research should prioritize these parameters to more comprehensively evaluate the impact of CVD on cognitive or movement dysfunction.

Whether CVD is a risk factor for PART, LATE, and ARTAG remains to be investigated; nevertheless, the possibility is quite important, given that CVD is a treatable or modifiable condition.^22^ The overall higher prevalence of CVD and associated ARTAG, LATE, and LBD, along with the reported higher CVD rate in northern regions of China, might also support this risk argument. Nevertheless, given the various subtypes of CVD, we further analyzed the associations between different subtypes of CVD and LATE as well as ARTAG. All types of CVD were associated with LATE with the associations between atherosclerosis and LATE, arteriolosclerosis and LATE, CAA and LATE persisted even after adjusting for age, sex, and Braak NFT stage, consistent with a recent report.^35^ But again, the underlying mechanisms require further investigation.

In our study, the positivity rate of TDP‐43 in the cortex is quite low, despite a relatively high rate in the limbic system. This suggests that LATE may be predominantly an age‐related disorder rather than a direct driver of FTD development, as suggested by Nelson et al.^36^ However, further research is needed in this area, especially given the less comprehensive clinical histories in our community‐based investigations. Additionally, we observed a positive association between LATE and ADNC, as reported by others,^35^, ^36^, ^37^, ^38^, ^39^ although the underlying biological connections require further investigation.

Community‐based study is likewise associated with several other caveats. For example, rare diseases, such as MSA, FTD, PSP, CBD, and AGD, are underrepresented. Additionally, unlike hospital‐based studies, as suggested above, the collection of clinical history, including medical management details, is much less comprehensive, making a diagnosis like CTE very challenging. These diseases will be a major focus in our next round of evaluation, which will use a much larger database expected to exceed 3000 brain donations in a few years (≈ 2027).

Besides community‐based autopsy issues, our investigation additionally has a few shortcomings. First, our sample predominantly consists of Han Chinese individuals sourced from major brain banks in China, with relatively few samples from ethnic minorities. This lack of ethnic diversity may limit the generalizability of our findings to the broader population. Second, we did not perform a detailed analysis of the relationship between the samples and clinical cognitive status or movement dysfunction, which could provide valuable insights into the correlation between neuropathological findings and cognitive outcomes or movement dysfunctions. Finally, we did not consider the apolipoprotein E (*APOE*) ε4 gene status, an important genetic factor known to influence AD risk, which may have implications for the interpretation of our results. Future research should address these limitations by including a more ethnically diverse sample, conducting thorough analyses of clinical cognitive correlations, and considering the impact of *APOE* ε4 gene status.

In conclusion, this largest community‐based autopsy study in China and Asia highlights the substantial prevalence and co‐occurrence of diverse neuropathologies, addressing a significant gap in current research. The association study also suggests that CVD might serve as a potential risk factor for all major neurological diseases, including PART, LATE, and ARTAG, providing valuable insights for future interventions targeting key brain diseases such as AD and PD.

## CONFLICT OF INTEREST STATEMENT

The authors declare no conflicts of interest. Author disclosures are available in the supporting information.

## CONSENT STATEMENT

All subjects provided informed consent.

## Supporting information



Supporting Information

Supporting Information
