# Supplementary material for: Prevalence of mixed neuropathologies in age‐related neurodegenerative diseases: A community‐based autopsy study in China
Source: Alzheimers Dement. 2024 Nov 25;21(1):e14369. doi: 10.1002/alz.14369 (PMC11782840; doi:10.1002/alz.14369)
Supplement: Supplementary file 1 — Supporting Information [file ALZ-21-e14369-s002.docx]

**Supplementary Table 1**. Assessment of the Altered Protein Expression

|  | H&E | HPτ | Aβ | αS | TDP43 |
| --- | --- | --- | --- | --- | --- |
| G.pre+ post central | X |  |  | X |  |
| Meninges | X |  | X |  |  |
| Hypothalamus | X |  |  |  |  |
| Temporal pole | X | X | X | X |  |
| Middle Frontal | X | X | X | X | X |
| Gyrus cinguli | X |  |  | X |  |
| Caudatum / Putamen | X | X | X |  |  |
| Putamen / Pallidum/ Ins. | X | X | X |  |  |
| Amy + ent. cort. | X |  |  | X | X |
| Hippocampus ant. | X | X | X |  |  |
| Hippocampus mid. | X | X | X |  | X |
| Hippocampus post. | X | X |  |  |  |
| Thalamus / subthal. | X |  |  |  |  |
| Lob. par. inf. (cerad) | X | X | X | X |  |
| Superior Parietal | X |  | X | X |  |
| Occipital pole | X | X | X |  |  |
| Substantia nigra | X | X | X | X |  |
| Colliculus inf. / pons | X |  |  |  |  |
| Loc. Coeruleus / pons | X |  |  | X |  |
| Cerebellum / dent. | X |  | X |  |  |
| Medulla oblongata | X |  |  | X |  |
| Spinal cord C | X |  |  |  |  |

Abbreviation: H&E: hematoxylin and eosin; HPτ: hyperphosphorylated-tau; Aβ: β-amyloid; αS: α-synuclein; p-TDP43: hyperphosphorylated transactive response DNA binding protein 43; Hip.A: Hippocampus Anterior; Hip.M: Hippocampus middle; Hip.P: Hippocampus Posterior; Men: Meninges; Par: Parietal; Occ: Occipital pole; LPI: Lob par. inf.; AE: Amy + ent. cort.; SN: Substantia nigra; MO: Medulla oblongata.

**Supplementary Table 2** Definitions of various cerebrovascular disease (CVD) pathology and the corresponding brain regions evaluated.

| Pathology | Definition | Brain areas assessed |
| --- | --- | --- |
| Atherosclerosis | Disease of medium-sized to large arteries at the base of the brain, characterized by formation of plaques showing varying degrees of destruction of the vessel wall and accumulation of lymphocytes and macrophages; in later stages plaques may contain necrotic core, cholesterol clefts and foci of calcification. | circle of Willis, basilar artery |
| Arteriolosclerosis | Hyaline thickening of walls of vessels <150 μm in diameter, not associated with lipid-containing cells replacing the tunica media. Diagnosis requires an absence of intramural inflammation, amyloid or fibrinoid necrosis. | All four lobes of cerebrum, Hippocampus, Basal ganglia, Thalamus |
| Intracerebral hemorrhage | Haemorrhagic lesion visible to the naked eye or found on microscopic examination; Large hemorrhage (Haemorrhagic lesion visible to the naked eye which is easily identifiable on macroscopic examination); Microhemorrhage [Haemorrhagic lesion (with parenchymal involvement) found on microscopic examination which is not visible  to the naked eye]. | All four lobes of cerebrum, Hippocampus, Basal ganglia, Thalamus |
| Cerebral infarction | Ischaemic lesion visible to the naked eye or found on microscopic examination; Large infarct (Maximum diameter >1 cm); Lacunar infarct (Cystic lesion visible to the naked eye but <1 cm in diameter); Microinfarct (Ischaemic lesion found on microscopic examination but not visible to the naked eye). | All four lobes of cerebrum, Hippocampus, Basal ganglia, Thalamus |
| cerebral amyloid angiopathy (CAA) | Amyloid was found to be deposited in the cerebral vascular wall | All four lobes of cerebrum, Hippocampus |

**Supplementary Table 3** Donor Demographics and Donation Information.

| Number | Brain Bank | Sex | Age at Death (y) | Cause of Death |
| --- | --- | --- | --- | --- |
| NO.01 | South Brain Bank | Male | 18 | Pneumonia, progressive muscular dystrophy |
| NO.02 | South Brain Bank | Male | 18 | Myasthenia Gravis |
| NO.03 | South Brain Bank | Male | 19 | Medulloblastoma of the Fourth Ventricle |
| NO.04* | South Brain Bank | Female | 19 | High-Grade Brainstem Glioma |
| NO.05* | South Brain Bank | Female | 19 | Drug Intoxication, Depression |
| NO.06 | South Brain Bank | Male | 20 | Respiratory Failure |
| NO.07 | South Brain Bank | Male | 21 | Epilepsy with Respiratory Failure |
| NO.08 | South Brain Bank | Male | 21 | Aplastic Anemia |
| NO.09 | South Brain Bank | Female | 21 | Cirrhosis with Hemorrhage |
| NO.10 | South Brain Bank | Male | 22 | Cerebral Palsy with Related Complications |
| NO.11 | South Brain Bank | Male | 22 | Asphyxia |
| NO.12 | South Brain Bank | Male | 22 | Drowning |
| NO.13 | South Brain Bank | Female | 22 | Osteosarcoma with Widespread Metastases |
| NO.14 | North Brain Bank | Male | 22 | Malignant Pericardial Tumor |
| NO.15 | North Brain Bank | Male | 22 | Epilepsy, Mental Retardation |
| NO.16 | South Brain Bank | Female | 23 | Colon Cancer with Widespread Metastases |
| NO.17 | North Brain Bank | Female | 23 | Brain Herniation |
| NO.18 | South Brain Bank | Male | 24 | Cerebral Palsy with Related Complications, Upper Gastrointestinal Ulcer with Hemorrhage |
| NO.19 | North Brain Bank | Female | 24 | Meningioma |
| NO.20 | South Brain Bank | Male | 25 | Complications Related to Epilepsy |
| NO.21 | South Brain Bank | Male | 25 | Brainstem Hemorrhage |
| NO.22 | North Brain Bank | Male | 26 | Lymphoma |
| NO.23 | South Brain Bank | Male | 27 | Schizophrenia with Related Complications |
| NO.24 | South Brain Bank | Female | 28 | Chondrosarcoma with Widespread Metastases |
| NO.25 | South Brain Bank | Male | 28 | Respiratory and Cardiac Arrest |

* The donor expressed their willingness to donate the brain during their lifetime. For the remaining cases, brain donation was made posthumously with joint consent from their legal guardians.

**Supplementary Table 4** Demographics of Brain Tissue Donors in northern brain banks

| Age Groups (years) | N | Gender | | Normal | ADNC | LBD | CVD | PART | LATE | ATARG | Others |
| --- | --- | --- | --- | --- | --- | --- | --- | --- | --- | --- | --- |
|  |  | M | F |  |  |  |  |  |  |  |  |
| ＜50 | 21 | 13(62) | 8(38) | 11(52) | 0(0) | 0(0) | 8(38) | 0(0) | 1(5) | 1(5) | 0(0) |
| 50-59 | 41 | 26(63) | 15(37) | 5(12) | 5(12) | 3(7) | 27(66) | 19(46) | 7(17) | 0(0) | 2(5) |
| 60-69 | 63 | 42(67) | 21(33) | 5(8) | 23(37) | 5(8) | 42(67) | 32(51) | 14(22) | 6(10) | 3(5) |
| 70-79 | 94 | 63(67) | 31(33) | 0(0) | 51(54) | 18(19) | 76(81) | 41(44) | 47(50) | 12(13) | 2(2) |
| 80-89 | 207 | 111(54) | 96(46) | 0(0) | 141(68) | 49(24) | 171(83) | 62(30) | 144(70) | 63(30) | 8(4) |
| ≥90 | 104 | 60(58) | 44(42) | 0(0) | 75(72) | 35(34) | 85(82) | 27(26) | 77(74) | 34(33) | 4(4) |
| All subjects  N (%) | 530 | 315(59) | 215(41) | 21(4) | 295(56) | 110(21) | 409(77) | 181(34) | 290(55) | 116(22) | 19(4) |

**Supplementary Table 5** Demographics of Brain Tissue Donors in southern brain banks

| Age Groups (years) | N | Gender | | Normal | ADNC | LBD | CVD | PART | LATE | ATARG | Others |
| --- | --- | --- | --- | --- | --- | --- | --- | --- | --- | --- | --- |
|  |  | M | F |  |  |  |  |  |  |  |  |
| ＜50 | 66 | 44(67) | 22(33) | 31(47) | 2(3) | 1(2) | 13(20) | 6(9) | 1(2) | 1(2) | 14(21) |
| 50-59 | 77 | 42(55) | 35(45) | 21(27) | 12(16) | 0(0) | 21(27) | 25(32) | 9(12) | 0(0) | 19(25) |
| 60-69 | 103 | 73(71) | 30(29) | 10(10) | 29(28) | 5(5) | 44(43) | 52(50) | 27(26) | 0(0) | 19(18) |
| 70-79 | 127 | 94(74) | 33(26) | 5(4) | 58(46) | 10(8) | 64(50) | 55(43) | 58(46) | 7(6) | 23(18) |
| 80-89 | 150 | 94(63) | 56(37) | 1(1) | 84(56) | 13(9) | 99(66) | 60(40) | 80(53) | 4(3) | 28(19) |
| ≥90 | 89 | 57(64) | 32(36) | 0(0) | 66(74) | 10(11) | 64(72) | 21(24) | 57(64) | 11(12) | 16(18) |
| All subjects  N (%) | 612 | 404(66) | 208(34) | 68(11) | 251(41) | 39(6) | 305(50) | 219(36) | 232(38) | 23(4) | 119(19) |

**Supplementary Table 6** Demographics of CVD subgroups in all brain banks.

| Age Groups (Years) | CVD | Atherosclerosis | Arteriosclerosis | Cerebral Hemorrhage | Cerebral Infarction | CAA |
| --- | --- | --- | --- | --- | --- | --- |
| ＜50 | 21(100) | 4(19) | 2(10) | 12(57) | 11(52) | 0(0) |
| 50-59 | 48(100) | 21(44) | 12(25) | 19(40) | 16(33) | 2(4) |
| 60-69 | 86(100) | 43(50) | 28(33) | 16(19) | 27(31) | 15(17) |
| 70-79 | 140(100) | 81(58) | 54(39) | 20(14) | 39(28) | 37(26) |
| 80-89 | 270(100) | 161(60) | 119(44) | 35(13) | 105(39) | 104(39) |
| ≥90 | 149(100) | 96(64) | 76(51) | 12(8) | 46(31) | 62(42) |
| All subjects  N (%) | 714(100) | 406(57) | 291(41) | 114(16) | 244(34) | 220(31) |

**Supplementary Table 7** Demographics of Intracerebral hemorrhage and Cerebral infarction.

| N (%) | Cerebral Hemorrhage | Large Hemorrhage | Microhemorrhage | Cerebral Infarction | Large Infarct | Lacunar Infarct | Microinfarct |
| --- | --- | --- | --- | --- | --- | --- | --- |
| All brain banks | 114(100) | 42(37) | 79(69) | 244(100) | 48(20) | 29(12) | 199(82) |
| Northern brain banks | 58(100) | 14(24) | 45(78) | 159(100) | 31(19) | 24(15) | 120(75) |
| Southern brain banks | 56(100) | 28(50) | 34(61) | 85(100) | 17(20) | 5(6) | 79(93) |

**Supplementary Table 8** Demographics of ADNC subgroups in northern brain banks

| Age Groups (years) | N | ADNC | ADNC (L) | ADNC (I) | ADNC (H) |
| --- | --- | --- | --- | --- | --- |
|  |  |  |  |  |  |
| ＜50 | 21 | 0(0) | 0(0) | 0(0) | 0(0) |
| 50-59 | 41 | 5(12) | 5(12) | 0(0) | 0(0) |
| 60-69 | 63 | 23(37) | 20(32) | 3(5) | 0(0) |
| 70-79 | 94 | 51(54) | 28(30) | 18(19) | 5(5) |
| 80-89 | 207 | 141(68) | 32(15) | 91(44) | 18(9) |
| ≥90 | 104 | 75(72) | 17(16) | 48(46) | 10(10) |
| All subjects (%) | 530 | 295(56) | 102(19) | 160(30) | 33(6) |

**Supplementary Table 9** Demographics of ADNC subgroups in southern brain banks

| Age Groups (years) | N | ADNC | ADNC (L) | ADNC (I) | ADNC (H) |
| --- | --- | --- | --- | --- | --- |
|  |  |  |  |  |  |
| ＜50 | 66 | 2(3) | 2(3) | 0(0) | 0(0) |
| 50-59 | 77 | 12(16) | 10(13) | 2(3) | 0(0) |
| 60-69 | 103 | 29(28) | 28(27) | 0(0) | 1(1) |
| 70-79 | 127 | 58(46) | 42(33) | 12(9) | 4(3) |
| 80-89 | 150 | 84(56) | 59(39) | 18(12) | 7(5) |
| ≥90 | 89 | 66(74) | 34(38) | 24(27) | 8(9) |
| All subjects (%) | 612 | 251(41) | 175(29) | 56(9) | 20(3) |

**Supplementary Table 10** Demographics of comorbidities in northern brain banks

|  | N (%) | Gender | | ADNC | LBD | CVD | PART | LATE | ARTAG |
| --- | --- | --- | --- | --- | --- | --- | --- | --- | --- |
|  |  | M | F |  |  |  |  |  |  |
| ADNC | 295 | 162(55) | 133(45) | 295(100) | 77(26) | 245(83) | 0(0) | 200(68) | 78(26) |
| LBD | 110 | 73(66) | 37(34) | 77(70) | 110(100) | 87(79) | 29(26) | 86(78) | 26(24) |
| CVD | 409 | 249(61) | 160(39) | 245(60) | 87(21) | 409(100) | 137(33) | 243(59) | 97(24) |
| PART | 181 | 121(67) | 60(33) | 0(0) | 29(16) | 137(76) | 181(100) | 83(46) | 36(20) |
| LATE | 290 | 168(58) | 122(42) | 200(69) | 86(30) | 243(84) | 83(29) | 290(100) | 86(30) |
| ARTAG | 116 | 69(59) | 47(41) | 78(67) | 26(22) | 97(84) | 36(31) | 86(74) | 116(100) |

**Supplementary Table 11** Demographics of comorbidities in southern brain banks

|  | N (%) | Gender | | ADNC | LBD | CVD | PART | LATE | ARTAG |
| --- | --- | --- | --- | --- | --- | --- | --- | --- | --- |
|  |  | M | F |  |  |  |  |  |  |
| ADNC | 251 | 155(62) | 96(38) | 251(100) | 23(9) | 177(71) | 0(0) | 143(57) | 16(6) |
| LBD | 39 | 27(69) | 12(31) | 23(59) | 39(100) | 23(59) | 13(33) | 20(51) | 5(13) |
| CVD | 305 | 205(67) | 100(33) | 177(58) | 23(8) | 305(100) | 86(28) | 154(50) | 19(6) |
| PART | 219 | 157(72) | 62(28) | 0(0) | 13(6) | 86(39) | 219(100) | 82(37) | 7(3) |
| LATE | 232 | 157(68) | 75(32) | 143(62) | 20(9) | 154(66) | 82(35) | 232(100) | 11(5) |
| ARTAG | 23 | 17(74) | 6(26) | 16(70) | 5(22) | 19(83) | 7(30) | 11(48) | 23(100) |


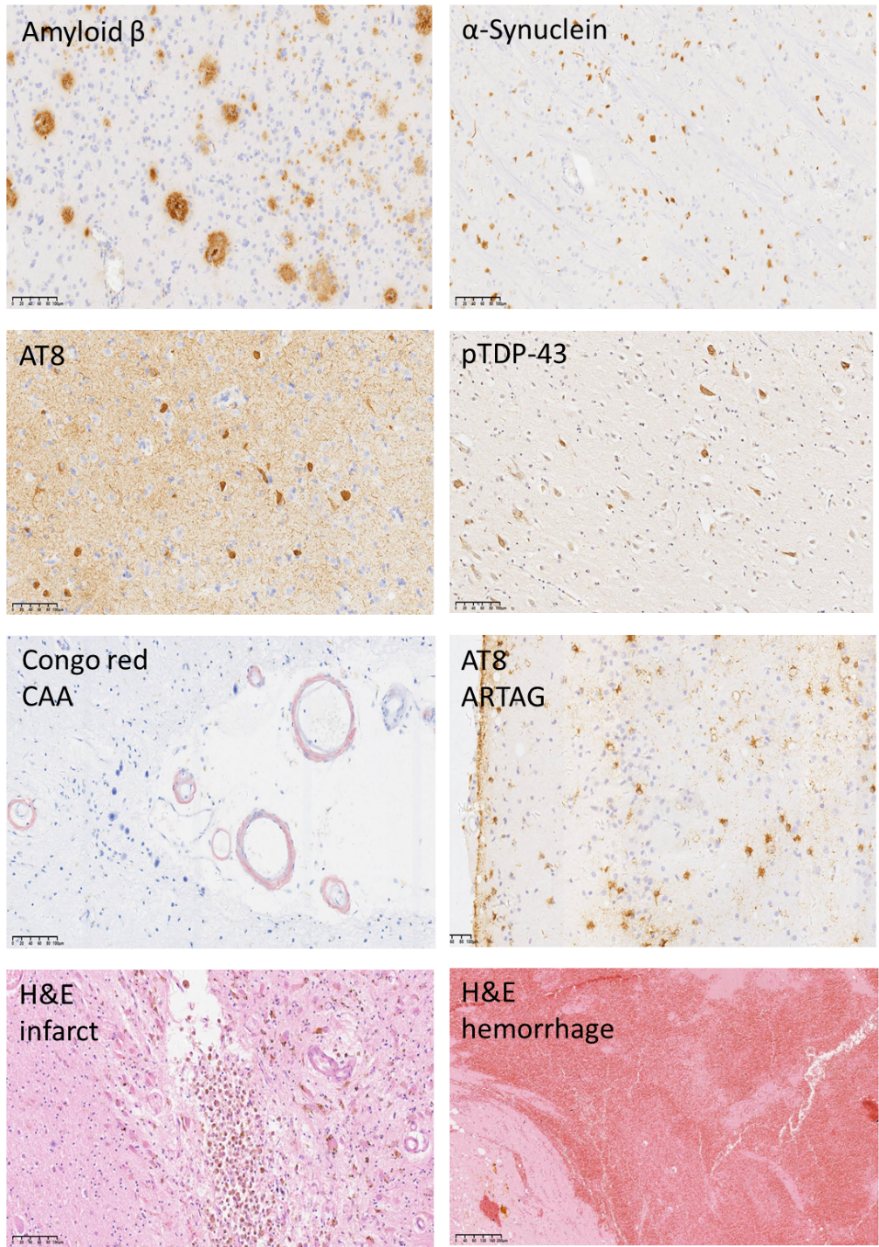


Supplementary Figure 1. Immunohistochemical detection of Amyloid b, AT8, a-Synuclein, and pTDP-43 and representative images of CAA, ARTAG, infarct, and hemorrhage.


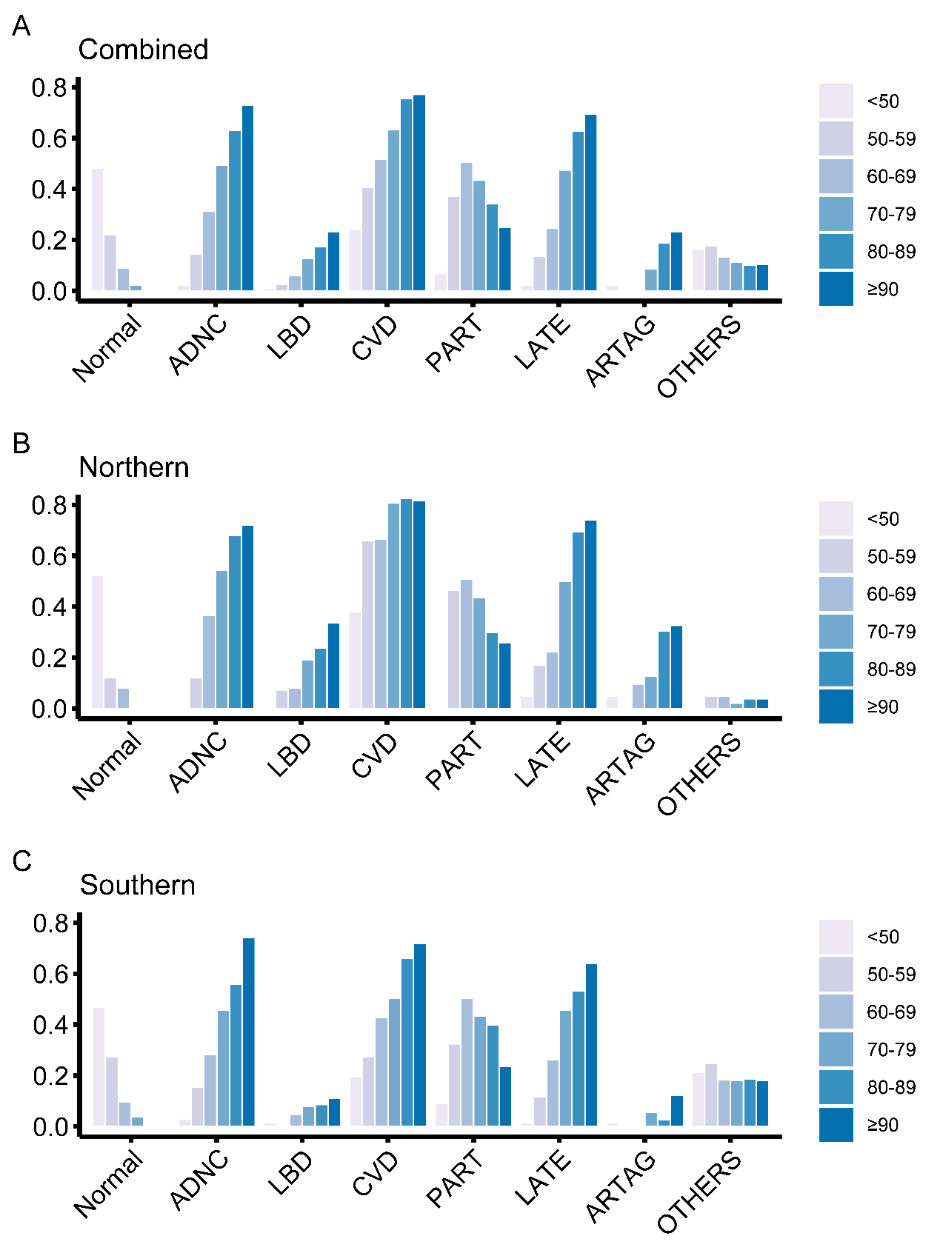


**Supplementary Figure 2. The prevalence of neuropathological conditions in each of the age groups. (A)** The prevalence of neuropathological conditions in all brains. **(B)** The prevalence of neuropathological conditions in brains from northern brain banks. **(C)** The prevalence of neuropathological conditions in brains from southern brain banks. The regional prevalence is detailed in **Supplementary Table 4** and **5**.


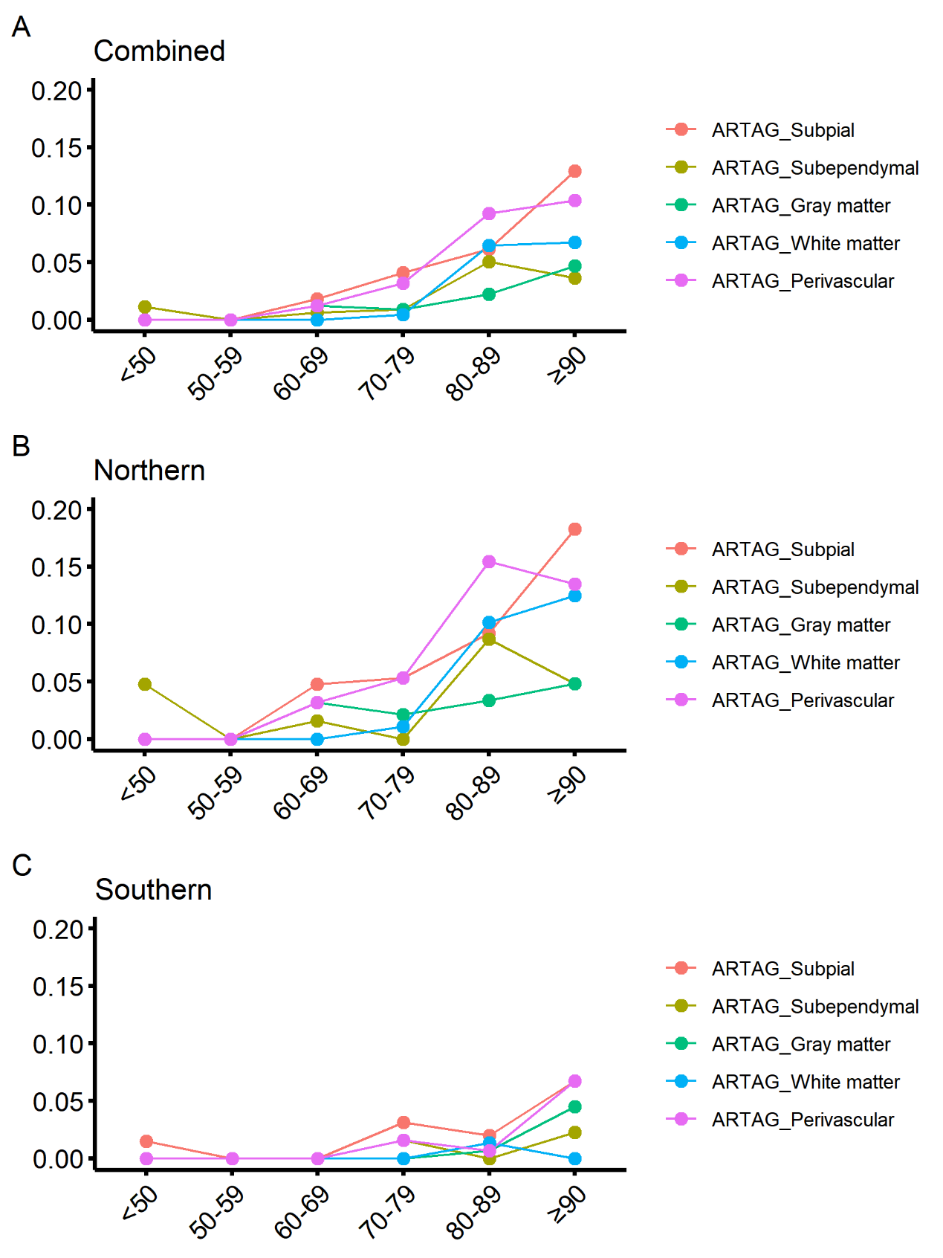


**Supplementary Figure 3.** **The prevalence of ARTAG subtypes in each of the age groups.** **(A)** The prevalence of ARTAG subtypes in each of the age groups in all brain banks. **(B)** The prevalence of ARTAG subtypes in each of the age groups in northern brain banks. **(C)** The prevalence of ARTAG subtypes in each of the age groups in southern brain banks.


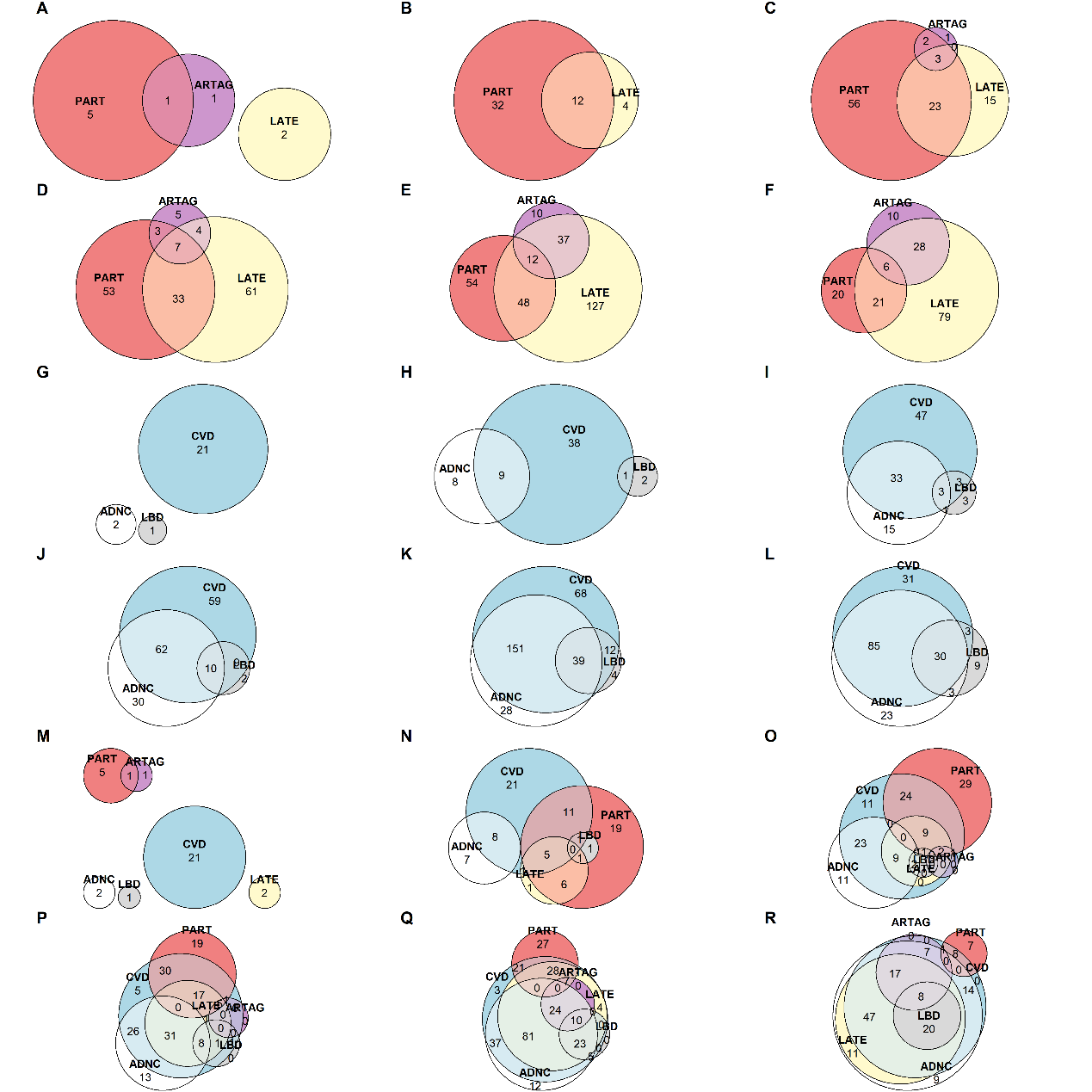


**Supplementary Figure 4. Venn diagrams illustrating the relationships among ADNC, LBD, CVD, PART, LATE, and ARTAG across different age groups.**

**(A-F)** Venn diagrams illustrating the relationships among PART, LATE, and ARTAG across different age groups. **(G-L)** Venn diagrams illustrating the relationships among ADNC, LBD, and CVD across different age groups. **(M-R)** Venn diagrams illustrating the relationships among ADNC, LBD, CVD, PART, LATE, and ARTAG across different age groups. **(A, G, M)** Donors younger than 50 years; **(B, H, N)** Donors aged 50–59 years; **(C, I, O)** Donors aged 60–69 years; **(D, J, P)** Donors aged 70–79 years; **(E, K, Q)** Donors aged 80–89 years; **(F, L, R)** Donors aged 90 years or older.


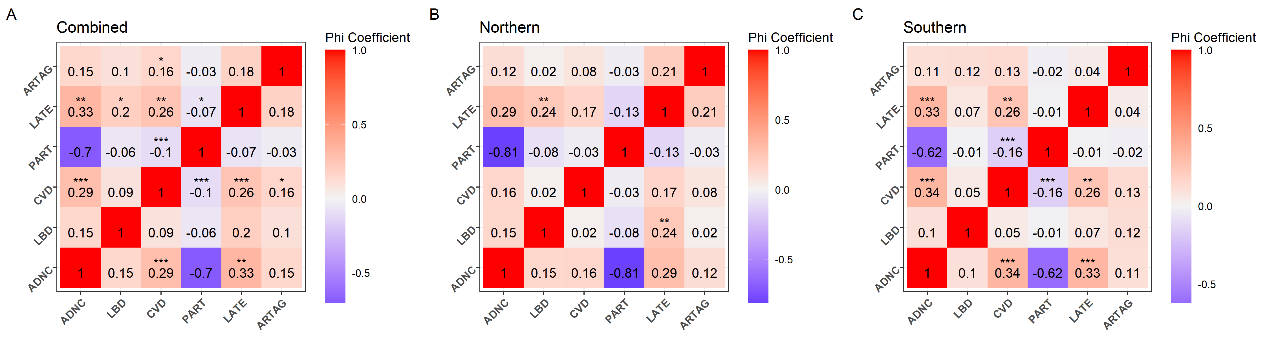


**Supplementary Figure 5. The association between age-related pathological states (including PART, LATE, and ARTAG) and diagnosis of ADNC, CVD, and LBD (covariates adjusted). (A)** The association between age-related pathological states and the diagnosis of **ADNC**, CVD, and LBD in all brain banks. **(B)** The association between age-related pathological states and the diagnosis of **ADNC**, CVD, and LBD in northern brain banks. **(C)** The association between age-related pathological states and the diagnosis of **ADNC**, CVD, and LBD in southern brain banks. The correlation among neuropathological conditions was represented by the Phi (φ) coefficient. The significance of the association was estimated by the logistic regression adjusting age, gender, and Braak NFT stage. ***, **, and * denote *P* < 0.001, *P* < 0.01, and *P* < 0.05, respectively.


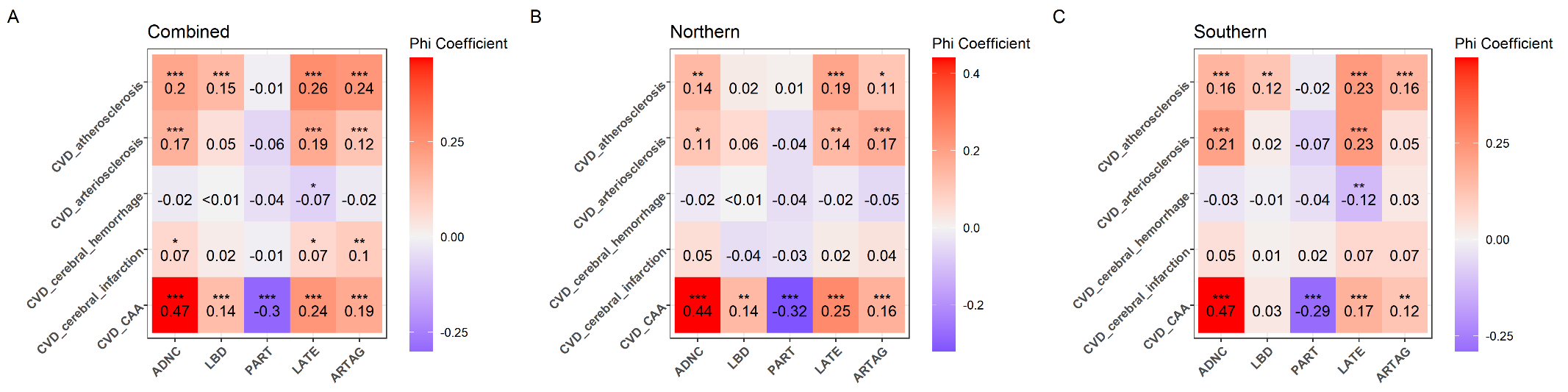


**Supplementary Figure 6. The association between CVD subtypes and the diagnosis of ADNC, LBD, PART, LATE and ARTAG (Chi-squared test, unadjusted). (A)** The association between CVD subtypes and the diagnosis of ADNC, LBD, PART, LATE and ARTAG in all brain banks. **(B)** The association between CVD subtypes and the diagnosis of ADNC, LBD, PART, LATE and ARTAG in northern brain banks. **(C)** The association between CVD subtypes and the diagnosis of ADNC, LBD, PART, LATE and ARTAG in southern brain banks. The correlation among neuropathological conditions was described by the Phi (φ) coefficient. The significance of the correlation was estimated by the Chi-squared test. ***, **, and * denote *P* < 0.001, *P* < 0.01, and *P* < 0.05, respectively.


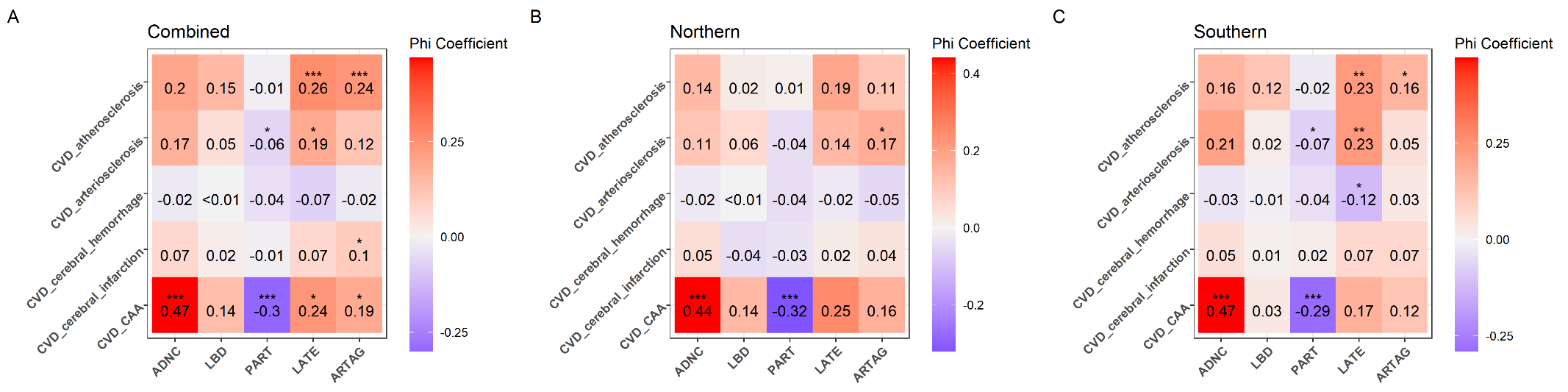


**Supplementary Figure 7. The association between CVD subtypes and the diagnosis of ADNC, LBD, PART, LATE and ARTAG (covariates adjusted). (A)** The association between CVD subtypes and the diagnosis of ADNC, LBD, PART, LATE and ARTAG in all brain banks. **(B)** The association between CVD subtypes and the diagnosis of ADNC, LBD, PART, LATE and ARTAG in northern brain banks. **(C)** The association between CVD subtypes and the diagnosis of ADNC, LBD, PART, LATE and ARTAG in southern brain banks. The correlation among neuropathological conditions was described by the Phi (φ) coefficient. The significance of the association was estimated by the logistic regression adjusting age, gender, and Braak NFT stage. ***, **, and * denote *P* < 0.001, *P* < 0.01, and *P* < 0.05, respectively.


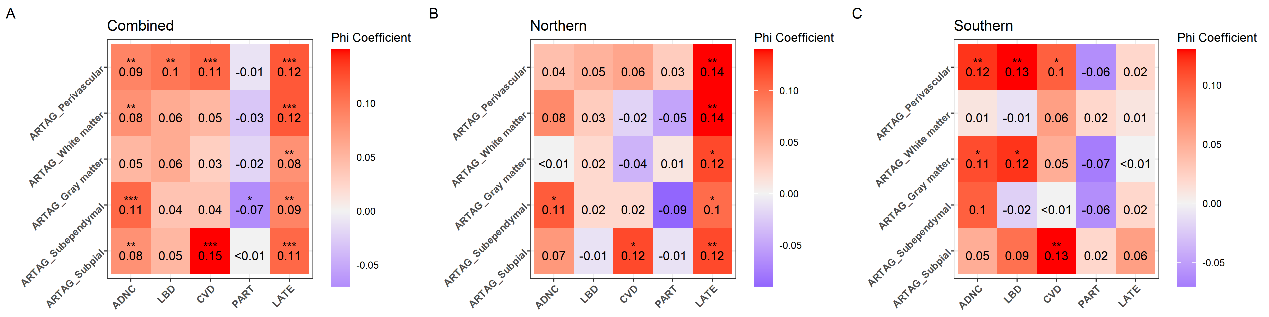


**Supplementary Figure 8. The association between ARTAG subtypes and the diagnosis of ADNC, LBD, CVD, PART, and LATE (Chi-squared test, unadjusted). (A)** The association between ARTAG subtypes and the diagnosis of ADNC, LBD, CVD, PART, and LATE in all brain banks. **(B)** The association between ARTAG subtypes and the diagnosis of ADNC, LBD, CVD, PART, and LATE in northern brain banks. **(C)** The association between ARTAG subtypes and the diagnosis of ADNC, LBD, CVD, PART, and LATE in southern brain banks. The correlation among neuropathological conditions was described by the Phi (φ) coefficient. The significance of the correlation was estimated by the Chi-squared test. ***, **, and * denote *P* < 0.001, *P* < 0.01, and *P* < 0.05, respectively.


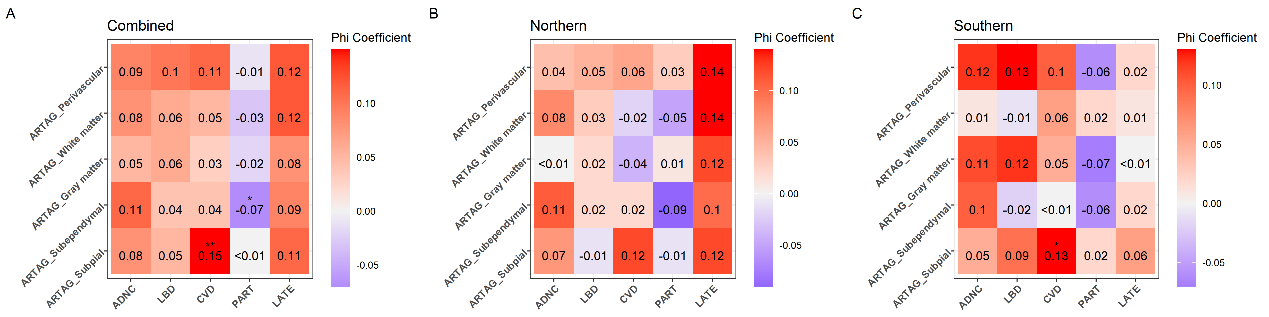


**Supplementary Figure 9. The association between ARTAG subtypes and the diagnosis of ADNC, LBD, CVD, PART, and LATE (covariates adjusted). (A)** The association between ARTAG subtypes and the diagnosis of ADNC, LBD, CVD, PART, and LATE in all brain banks. **(B)** The association between ARTAG subtypes and the diagnosis of ADNC, LBD, CVD, PART, and LATE in northern brain banks. **(C)** The association between ARTAG subtypes and the diagnosis of ADNC, LBD, CVD, PART, and LATE in southern brain banks. The correlation among neuropathological conditions was described by the Phi (φ) coefficient. The significance of the association was estimated by the logistic regression adjusting age, gender, and Braak NFT stage. ***, **, and * denote *P* < 0.001, *P* < 0.01, and *P* < 0.05, respectively.
